# Limitations and challenges in the characterization of extracellular vesicles from stem cells and serum

**DOI:** 10.1007/s00604-025-07147-4

**Published:** 2025-04-21

**Authors:** Sara Escudero-Cernuda, Noemi Eiro, María Fraile, Francisco J. Vizoso, Belén Fernández-Colomer, María Luisa Fernández-Sánchez

**Affiliations:** 1 Department of Physical and Analytical Chemistry, University of Oviedo, Avda. Julián Clavería, 8, 33006 Oviedo, Asturias, Spain; 2 Research Unit, Jove Hospital Foundation, Avda. Eduardo Castro, 161, 33920 Gijón, Asturias, Spain; 3 Service of Neonatology, Department of Pediatrics, Hospital Universitario Central de Asturias, Oviedo, Spain

**Keywords:** Human uterine cervical stem cells, Mesenchymal stem cells, Extracellular vesicles, Exosomes, Particle characterization

## Abstract

Exosomes are a subpopulation of nanosized extracellular vesicles (EVs), formed by a lipid bilayer and naturally secreted by cells. They transport RNA, microRNAs, bioactive proteins, and lipids and play an important role in intercellular communication. Exosomes are a promising alternative cell-free therapy in regenerative medicine, immunotherapy, and drug delivery. The implementation of new exosomes treatments requires knowledge of its concentration, purity, and full characterization. However, comparing different studies is highly challenging due to the lack of validated methodologies for isolation and determination, as well as a lack of well-characterized exosomes reference standards. In this work, human uterine cervical mesenchymal stem cell (hUCESC) EVs have been isolated by ultracentrifugation and characterized, discussing the current limitations of characterization methods. First, total protein assays are heavily influenced by free-protein and lipid contaminations which confirm the need of employing several methods for vesicular protein determination. This purity variation seems heavily influenced by the vesicles origin as more complex mediums originate more matrix interferences. Size exclusion high performance liquid chromatography has been demonstrated as a new methodology for purity assessment of hUCESC-EVs and commercial EVs (adipose stem cells and human serum). The results found low purity in the commercial exosomes highlighting that protein and lipid purity must be included in the commercial EVs. Finally, the combination of this chromatography method with total protein assays proved that particle concentration could be estimated using vesicular protein concentration.

**Graphical abstract:**

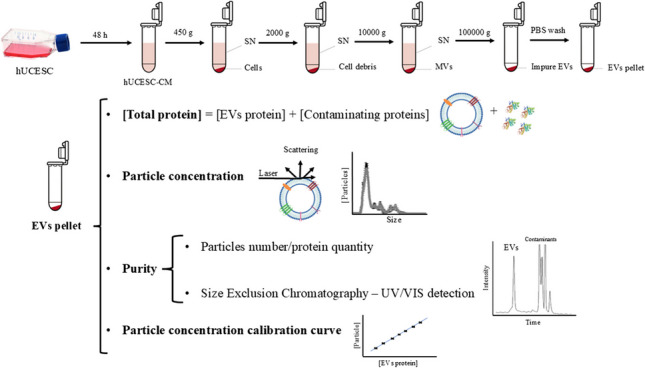

**Supplementary Information:**

The online version contains supplementary material available at 10.1007/s00604-025-07147-4.

## Introduction

Mesenchymal stem cells (MSC) are non-hematopoietic, multipotent, self-renewable cells that can differentiate into several cell lineages [1, 2]. MSC have emerged as a promising therapeutic strategy because of their anti-inflammatory, antimicrobial, anti-oxidative stress, and regenerative capacities [2, 3] which can be ascribed to the soluble factors (immunomodulators, cytokines, chemokines, and growth factors) and extracellular vesicles (EVs) released by them [4–6]. The variety of molecules and EVs released by the MSC into the cell culture media constitutes the secretome or conditioned media (CM) [7].

Therapeutic applications using EVs, instead of the whole secretome, are an emerging research area in regenerative medicine [8]. This is because EVs have a significant role in intercellular communication and signaling, in both physiological and pathological processes [9, 10]. MSC-derived EVs (MSC-EVs) exert their functions through the transfer of their cargo (proteins, lipids, metabolites, and diverse nucleic acids) to target cells [11]. These EVs are classified according by their size and origin into “large extracellular vesicles” (lEVs) for traditional apoptotic bodies and microvesicles (more than 200 nm) and the term “small extracellular vesicles” (sEVs) for exosomes (below 200 nm) [12]. Among EVs, exosomes are of special interest due to their ability to reproduce the therapeutic effects of MSC as they carry cell components which reflect the cell metabolic state. Exosomes are lipid bilayer “nanoparticles” in the size range around 50–150 nm with the presence of tetraspanins (CD9, CD63, and CD81), Alix and TSG101 as specific biomarkers [13].

In this sense, MSC-EVs constitute a promising alternative as cell-free therapy and therapeutic drug nanocarriers [14]. However, its potential application requires reproducible isolation, enrichment, and physical and (bio)chemical characterization. It has been previously reported that there is a general lack of proper EV isolation and characterization methodologies [15], probably due to its nano size, heterogeneity, biological origin, and the inability to separate them from other components like liposomes or contaminant proteins. To obtain reliable and reproducible functional assays, it is important to control the particle concentration (particles mL^−1^) and vesicular purity (vesicle concentration/protein concentration). One common pitfall is to assume that total protein content corresponds only to EVs proteins [16]. Therefore, choosing total protein to quantify vesicle concentration is not always an accurate approximation. In addition, nanoparticle tracking analysis (NTA), considered the golden standard for EV quantification, has some drawbacks due to its principles based on light scattering. Vesicles under 50 nm are not quantified by this technique [17, 18]; meanwhile, microvesicles or protein aggregates, which also scatter light, could impact heavily the EV quantification [19]. Because of these limitations, researchers have worked to develop new methodologies for exosome isolation, characterization, and quantification to improve the quality of the exosomes samples and the validity of the results of their biomedical applications.

Recent studies have demonstrated the therapeutic potential of human uterine cervical stem cell–conditioned media (hUCESC-CM), including antitumoral effect [9], corneal regeneration capacity [10], antifungal activity [11], and anti-inflammatory effect [20]. The understanding of the significant roles of EVs produced from hUCESC, which make them essential as therapeutic agents, requires an exhaustive characterization and knowledge of their composition.

In this work, hUCESC-EVs were isolated by differential ultracentrifugation and physically characterized by NTA, transmission electron microscopy (TEM), total proteins assays, and high-performance size exclusion chromatography (HPLC-SEC). Limitations on characterization methodologies for both hUCESC-EVs and commercial EVs (adipose tissue MSCs and human serum) and the significance of “purity” determination were discussed. Also, the combination of this chromatography with total protein assays proved that particle concentration could be estimated using vesicular protein concentration. Finally, flow cytometry (FC) was used to determine the presence of exosomes by detecting its specific biomarkers, CD9 and CD81, and to study the vesicle membrane integrity using a calcein-violet fluorescent stain. These methodologies were applied to compare the characteristics of EVs isolated from different batches of hUCESC donors and commercial EVs from stem cells and serum.

## Materials and methods

### Ethics statement

The Regional Clinical Research Committee of the Principality of Asturias (“Comité Ético de Investigación Clínica Regional del Principado de Asturias”) has approved this study (ref. 99/18, 21 March 2018), and it complied with all applicable national legislation. hUCESC were obtained using cervical tissue from women who underwent routine gynecological exams or hysterectomy at Fundación Hospital de Jove in Asturias, Spain. All donors have signed the informed consent forms.

### hUCESC cell culture and CM harvest

hUCESC were cultured in cell stacks (Corning, NY) at 3000 cells/cm^2^ in DMEM-F12 with 10% fetal bovine serum (FBS), penicillin, and streptomycin (Corning) in an air-CO_2_ (95–5%) atmosphere at 37 °C in a Celsius 2007 cell incubator (Memmert GmbH, Germany). The cells were grown until 80% of cell confluence, cell culture media was discarded, and cells were washed three times with phosphate-buffered saline (PBS) (Lonza, Spain). Then, hUCESC were cultured 48 h in DMEM-F12 without phenol red (Corning), and the conditioned media from hUCESC (hUCESC-CM) was harvested and centrifugated 5 min at 450 g for cell removal.

### Extracellular vesicle isolation from hUCESC-CM

hUCESC-EVs were isolated by a differential centrifugation protocol at 4 °C. Briefly, the conditioned media was centrifugated at 2000 g, 10 min to remove cell debris, and the supernatant was centrifuged 30 min at 10,000 g to remove apoptotic bodies and microvesicles on a centrifuge 1580R (Gyrozen, South Korea). Then, the supernatant was transferred to ultracentrifuge tubes (38.5-mL Open-Top Thinwall Polypropylene Tubes) and ultracentrifuged at 100,000 g, 70 min on an Optima L- 90 K Ultracentrifuge (Beckman Coulter Inc., USA) to precipitate the EVs. The resultant pellet was washed with PBS followed by a second step of ultracentrifugation at the same conditions. The EVs were resuspended in 0.22-µm filtered PBS with sucrose at 1% (Sigma-Aldrich, USA) at a final volume of 200 µL and frozen at − 80 °C. The hUCESC-EV samples obtained from five donors were labelled as hUCESC 1–5, whereas those from different CM isolations of the same donor were designed with letters A to C.

Commercial exosomes “standards” (as stated by the commercial house data sheet) from human adipose MSC (batches 250,419 and 091221), and human serum (batches 110,620 and 101,022) were purchased from HansaBioMed Life Sciences (HansaBioMed, Estonia). Human serum EVs were analyzed to study how a more complex matrix will impact the characterization methodologies. The commercial EVs were prepared according to the manufacturer protocol and diluted with PBS to obtain the desired particle concentration. The different batches were labelled 1 and 2. This commercial EVs were purified by a combination of tangential flow filtration (TFF) and SEC and are stated as “purified” and characterized by NTA (particle concentration and size distribution) and FC (CD9, CD63, CD81) [21]. The purity and characterization of this commercial EVs will be discussed in the results and discussion sections.

### Size distribution and particle concentration

The sample particle size distribution was performed by dynamic light scattering (DLS) on a Zetasizer Nano Series (Malvern Panalytical, UK) and/or by NTA using a Nanosight LM10 (Malvern Panalytical, UK) equipped with a 405-nm violet laser and a camara level of 15. Samples were diluted in 0.22-µm filtered PBS to achieve a concentration within the optimal NTA analysis range (1×10^6^ to 1×10^9^ particles mL^−1^). The samples were measured at four different dilutions for inter-assay studies. Each dilution was captured three times (3 × 30 s acquisition) for the intra-assay assessment. The resulting videos were analyzed with NTA software version 3.1 (detection threshold 5). Median values for concentration and size distribution were calculated.

### Transmission electronic microscopy (TEM)

EV samples were diluted with 0.22-µm filtered PBS, fixed 15 min with 2% paraformaldehyde, and dyed 1 min with 2% phosphotungstic acid. Finally, samples were dried and visualized with a transmission electronic microscopy JEM- 1011 (JEOL, Japan) at 100 kV.

### Total protein concentration

Total protein concentration was determined with a Pierce™ Bicinchoninic Acid Assay kit (Thermo Fisher Scientific, USA) and a Bradford reagent 5x × (SERVA, Germany). The calibration graphs between 0 and 60 µg mL^−1^ were performed with a bovine serum albumin (BSA) standard (Thermo Fisher Scientific, USA) in PBS. Both assays were performed in Nunc™ 96-well immuno plate (Thermo Fisher Scientific, USA) and the absorbance at 562 nm for BCA and 595 nm for Bradford was measured in a microplate reader Multiskan SkyHigh (Thermo Fisher Scientific, USA).

### Purity assessment by high-performance size exclusion chromatography (HPLC-SEC-UV/VIS)

A Size Exclusion Column Superose 6 Increase 10/300 GL (Cytiva, USA) with exclusion limit 5–5000 kDa was selected to evaluate the purity of the EV samples. The chromatographic separation was performed with an HPLC Agilent 1100 series pump and UV/VIS detector (Agilent, USA). The mobile phase consisted of phosphate-buffered saline solution (0.2 g L^−1^ KCl, 0.2 g L^−1^ KH_2_PO_4_, NaCl 8 g L^−1^, and 1.15 g L^−1^ Na_2_HPO_4_ (Sigma-Aldrich, USA)) filtered through a 0.1 µm PVDF membrane filter (Merck KGaA, Germany) at a flow rate of 1 mL min^−1^.

The column calibration was performed with BSA, cytochrome C, thyroglobulin (Sigma-Aldrich, USA), and a rabbit IgG antibody (Bioss, USA).

### Exosome biomarker detection and membrane integrity by flow cytometry

Exosomes were specifically detected with two antibodies: anti-CD9 antibody marked with FITC (Abcam, UK) and anti-CD81 antibody marked with PE (Bio-Rad, USA) both at 0.5 µg mL^−1^. Also, for vesicle integrity, Molecular Probes™ CellTrace™ Calcein Violet, AM (Invitrogen, USA) at 20 µM was used. The EV samples were incubated at a vesicle concentration between 1×10^7^ to 1×10^9^ vesicles mL^−1^ for 30 min in 0.1 µm filtered PBS and room temperature. Then, the samples were diluted 1:1 in 0.1 µm filtered PBS, and the fluorescence was monitored by a CytoFLEX S Flow Cytometer using a Violet Laser (405 nm) and its light side scattering (SSCviolet). The SSCviolet, FITC, and PE fluorescence channel detector gain and SSCviolet threshold were previously optimized using Megamix-Plus SSC fluorescent Beads (Cosmo Bio, USA) which consists of four different-sized beads (500, 240, 200, and 160 nm) with Fluorescein-5-isothiocyanate as a fluorescent biomarker. The gated events were found to be within the EVs’ size range by comparison to the size calibration beads.

### Statistical analysis

Data analysis and statistics (*F*-test for variances, ANOVA, and Kruskal Wallis) were conducted with OriginPro 2018, and a *p* value < 0.05 was considered statistically significant.

## Results

### Size distribution and morphology analysis

NTA was used to calculate the size distribution of particles in the EV samples with sizes between 50 and 1000 nm [17, 18]. First, quality control was performed by measuring a monodisperse polystyrene bead standard of 100 nm, obtaining a size of 99 ± 1 nm. EV samples were prepared by appropriate dilution (20 to 100 particles per microscopic field). Size distribution showed a polydisperse distribution with a maximum peak around 100–150 nm (Fig. S1). As can be seen in Table 1, no differences in size distribution were found depending on the EVs origin (serum, MSC from adipose tissue or hUCESC) and between batches (hUCESC 2 A and 2B and adipose MSC 1 and 2). As one sample size distribution was not parametric, a Kruskal Wallis test was performed, and no differences were found in size distribution (*p* = 0.071). The percentage of particle sizes compatible with exosome (50–150 nm) varies between 49 and 76% regardless of MSC origin (hUCESC, adipose, or serum).

**Table 1:** NTA size distribution median (nm) and percentage of EVs between 50 and 150 nm present in hUCESC EVs and commercial EVs (*n* = 3)

*EVs sample*	*Median* ± *SD (nm)*	*% particles 50–150 nm*
*hUCESC 1*	122 ± 3	76 ± 4
*hUCESC 2-A*	121 ± 7	64 ± 9
*hUCESC 2-B*	143 ± 3	53 ± 4
*hUCESC 3*	145 ± 8	57 ± 14
*hUCESC 4*	132 ± 4	63 ± 9
*hUCESC 5*	134 ± 19	59 ± 16
*Adipose MSC 1*	137 ± 3	57 ± 5
*Adipose MSC 2*	151 ± 10	49 ± 15
*Serum 1*	118 ± 3	74 ± 7

**Fig. 1:**
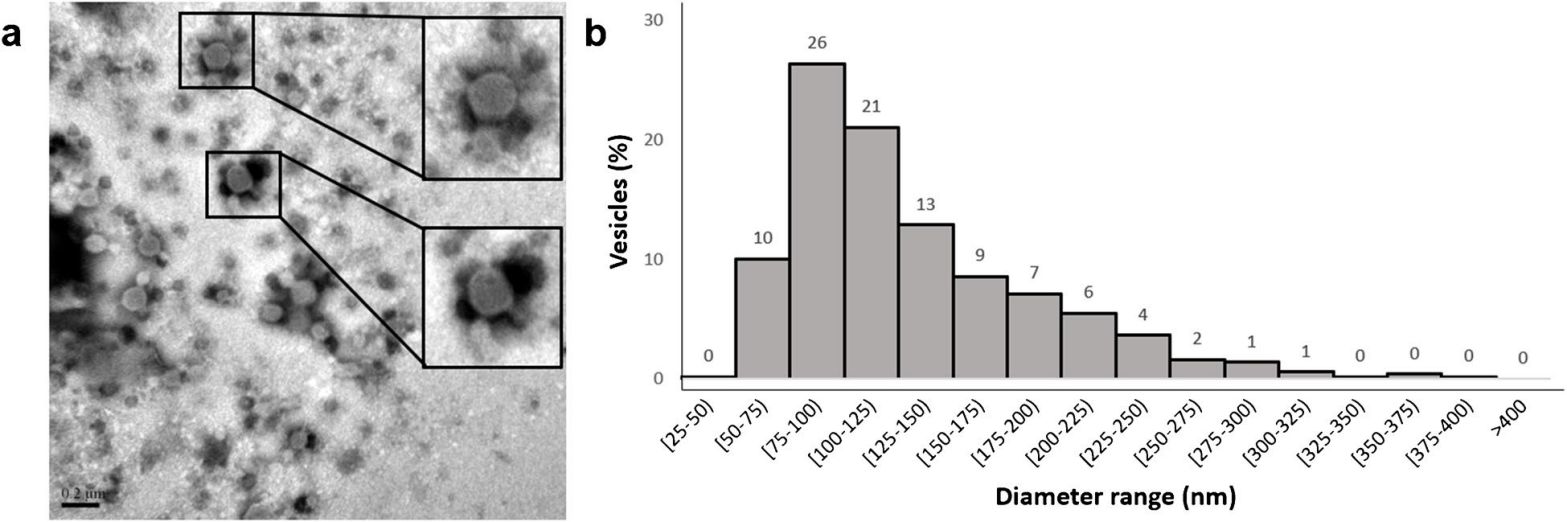
Transmission electron microscopy results of hUCESC 5. **a** Representative micrography. Scale 200 nm. **b** Relative size distribution histogram (*n* = 489)

The EV samples were also visualized by TEM after staining with phosphotungstic acid. TEM results have shown that exosomes have a spherical structure (Fig. 1a), and their relative size distribution histogram (489 vesicles measured) is shown in Fig. 1b. As can be seen, most EV sizes were found between 50 and 150 nm (> 70%) with a maximum peak corresponding to the 75–100-nm range. This decrease in size is explained as follows: while NTA measures the hydrodynamic diameter, TEM measures the true diameter of the particle. TEM images are summarized in Fig. S2.

### NTA particle concentration: repeatability, reproducibility, and accuracy

NTA is the most used method to measure particle size and concentration of small particles in biological samples. This study evaluates the intra- and inter- assay variations of NTA for adipose MSC and hUCESC samples.

The EV samples were vortexed and diluted in 0.22 µm filtered PBS to a concentration within the recommended concentration measurement range (1×10^6^ to 1×10^9^ particles mL^−1^). For each sample dilution, three 30s videos were recorded in identical instrument-optimized settings. The intra-assay variation of the median vesicle size (nm) and concentration (particles mL^−1^) were calculated by analyzing the same sample sequentially (*n* = 3) at several dilutions (see Table 2). As can be seen, the intra-assay precision obtained for adipose MSC and hUCESC samples is similar, with a relative standard deviation (RSD) between 1 and 11% depending on the dilution and a median size always between 100 and 150 nm. The concentration inter-assay variation was calculated by analyzing four different dilutions of the EV samples. Significant differences in particle concentration RSDs (see Table 2) were observed for the commercial adipose MSC EVs with a RSD of 43% across the tested dilutions. This inter-assay variation was also higher to the 15% and 18% obtained for hUCESC 1 and 2-A, respectively. A *F* test for variance study was performed, and the inter-assay variation of MSC 1 was found statistically higher than the variation for hUCESC samples.

**Table 2:** NTA results: dilution in chronological order, particle concentration, size distribution, intra-assay and inter-assay variation studies for commercial EVs from adipose MSC 1 (NTA commercial value = 4.4×10^11^ particles mL^−1^), and hUCESC-EV samples

*EV sample*	*Dilution*	*Concentration (p/mLx10*^*11*^*)*	*Intra-assay RSD (%)*	*Inter-assay RSD (%)*	*Median size (nm)*
*Adipose MSC 1*	1:200	4.08 ± 0.18	4	43	137 ± 3
	1:2000	11.2 ± 0.1	5		125 ± 3
	1:100	7.62 ± 0.32	4		142 ± 19
	1:500	5.80 ± 0.63	11		116 ± 3
*hUCESC 1*	1:200	1.51 ± 0.10	6	15	122 ± 3
	1:100	1.52 ± 0.06	4		129 ± 6
	1:100	1.43 ± 0.07	5		117 ± 4
	1:68	1.94 ± 0.08	3		147 ± 4
*hUCESC 2-A*	1:100	0.98 ± 0.06	6	18	135 ± 3
	1:100	0.75 ± 0.07	10		121 ± 7
	1:40	0.64 ± 0.01	2		122 ± 2
	1:64	0.73 ± 0.01	1		130 ± 2

The NTA accuracy was determined analyzing the adipose MSC EVs and comparing with the “true value” given by the manufacturer (see Table 2) which is 4.4×10^11^ particles mL^−1^. Relative errors obtained with different dilutions ranged between − 7 and 155%, and the highest relative errors correspond to dilutions 1:100 and 1:2000 (lower and higher dilutions tested), rather than possible lysis and/or aggregation due to freeze and thaw cycle suffered by the commercial samples [22].

### Total protein content and purity assessment

One of the most used methods to quantify EVs is the determination of total protein concentration via colorimetric assays. These assays quantify not only vesicular protein (EV-protein) but also the contaminant proteins co-isolated with the EVs and other types of interferences depending on the assay carried out. As BCA and Bradford assays possess different interferences and BCA is heavily interfered by lipids/lipoproteins, the hUCESC 4 and serum 2 total protein contents were analyzed with both assays. Meanwhile, the total protein content was the same for hUCESC 4 (30 ± 2 µg mL^−1^ with BCA and 26 ± 3 µg mL^−1^ with Bradford); in the case of serum 2, the protein determined by BCA and Bradford was 1010 ± 43 µg mL^−1^ and 224 ± 31 µg mL^−1^, respectively. The results could indicate the presence of lipids and/or lipoproteins in the commercial serum EVs which cause great interferences in the BCA assay. Because of this, Bradford assay was performed for commercial EVs; meanwhile, BCA assay was selected for hUCESC analysis due to its greater sensibility. As can be seen in Table 3, the total protein measurements in the hUCESC-EV samples ranged between 12 and 31 µg mL^−1^ which is more than 10 times lower than in both commercial exosomes. As impurity problems could be related to these disparities, hUCESC samples and commercial EVs were further studied.

**Table 3:** Total protein concentration, particle concentration and purity calculated in particles per total protein quantity for hUCESC-EV samples and commercial EVs. Unpure < 1.5×10^9^ particles µg protein^−1^ [24]

*EV sample*	*Total protein concentration (µg mL*^*−1*^*)*	*Particle concentration (p/mL·10*^*11*^*)*	*Particles/µg total protein (p/µg·10*^*9*^*)*
*hUCESC 1*	31.0*	1.51	5.16
*hUCESC 2-A*	16.0*	0.98	4.69
*hUCESC 2-B*	12.3*	1.01	8.21
*hUCESC 3*	13.6*	0.65	4.77
*hUCESC 4*	29.7*	0.99	3.32
*hUCESC 5*	14.3*	1.34	9.37
*Adipose MSC 1*	579	4.10	0.71
*Adipose MSC 2*	657	8.13	1.24
*Serum 1*	602	42.8	7.11
*Serum 2*	224	30.0	13.4

*Total protein concentration≅vesicular protein concentration

One simple approach for estimating the purity of EVs is to consider that 1 µg of total protein is equivalent to 1×10^9 ^– 1×10^10^ particles [23]. Another one is to quantify vesicle purity by the particle to protein ratio (particles (p)/µg protein^−1^). According with Webber and Clayton’s criteria [24], the EV preparations with ratios > 3×10^10^ particles µg^−1^ protein are highlighted as high purity and those < 1.5×10^9^ particles µg^−1^ protein are considered impure. As shown in Table 3, the hUCESC-EV preparations present ratios in the range of 5×10^9^ to 9×10^9^ particles µg^−1^ protein which can be considered pure according with Webber and Clayton’s criterium. It also must be highlighted that the purity of hUCESC samples is similar to those found in serum commercial EVs and significantly superior to the purity of the two commercial samples from adipose MSC, which are not considered pure by this criterium (< 1.5×10^9^ particles µg^−1^ protein). These findings could indicate the presence of contaminants.

**Fig. 2:**
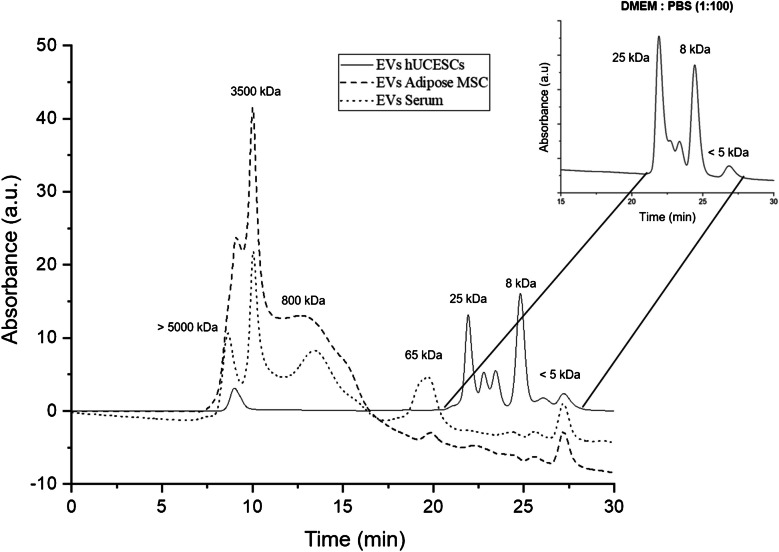
HPLC-SEC chromatograms for EV hUCESC 5, commercial EVs from adipose MSC 2, and commercial EVs from human serum 1. The signals from 20 min in hUCESC-EVs seem to come from DMEM-F12 remnants

As Webber and Clayton’s principle found that adipose MSC EVs were impure, a new purity assessment methodology was developed by HPLC-SEC with UV/VIS detection. The column calibration was made by the analysis of protein standards, and the calibration curve was logM =  − 0.1786 *t*_r_ + 8.3327, where *M* is the molecular weight and *t*_r_ the retention time. The chromatogram profiles obtained for EV samples are shown in Fig. 2. EVs from hUCESC 5 have two remarkable zones in the chromatogram. The peak at 9 min was identified as the EV peak while the other peaks after 20 min corresponded to low molecular weight compounds (< 25 kDa) which are also present in the chromatogram of DMEM-F12 culture media. Because DMEM-F12 is a synthetic protein-free medium, these peaks were not quantified by the BCA protein assay. Meanwhile, both commercial exosomes (adipose MSC 2 and serum) present different SEC profiles. Apart from the EV peak at around 9–10 min, the additional peaks at high molecular weights (3500 kDa and 800 kDa) could be protein aggregates, large protein complexes, or non-vesicular particles like lipoproteins. Also, the signal around 65 kDa detected in commercial serum exosomes could be due to serum albumin. The first two peaks from adipose MSC 1 were collected and analyzed by DLS to corroborate that peak 1 (9.8 min) is the only one composed by vesicles with an average diameter of 62 ± 35 nm which correlates with small EVs. However, vesicles were not present in peak 2 (10.7 min). This means that only peak 1, which is 24% of the total signal, contributes to the vesicular protein signal. Similar procedure was applied to serum 1 sample obtaining a percentage of 22% for peak 1. Then, vesicular protein concentrations were calculated considering the purity of the vesicle preparations (total protein × purity percentage).

hUCESC-EV chromatogram shows a well-separated EV peak from the low molecular compounds corresponding to remnants of the culture media. However, chromatograms of both commercial exosomes present greater contaminations by large proteins and proteins aggregates which are more evident in adipose MSC. The signals at lower retention times could also be explained by co-isolation of lipoproteins from adipose tissue and serum [16, 25]. More chromatograms of commercial serum exosomes and hUCESC-EVs are summarized in Fig. S3.

### Biomarker and integrity assessment by flow cytometry

Flow cytometry offers a multiparametric technology for EV counting and phenotyping. Here, two specific membrane exosomal biomarkers (CD9 and CD81) were used to identify the presence of exosomes. The EV detection threshold and gains were previously optimized with Megamix-Plus SSC FITC-fluorescent Beads (Fig. S4).

**Fig. 3:**
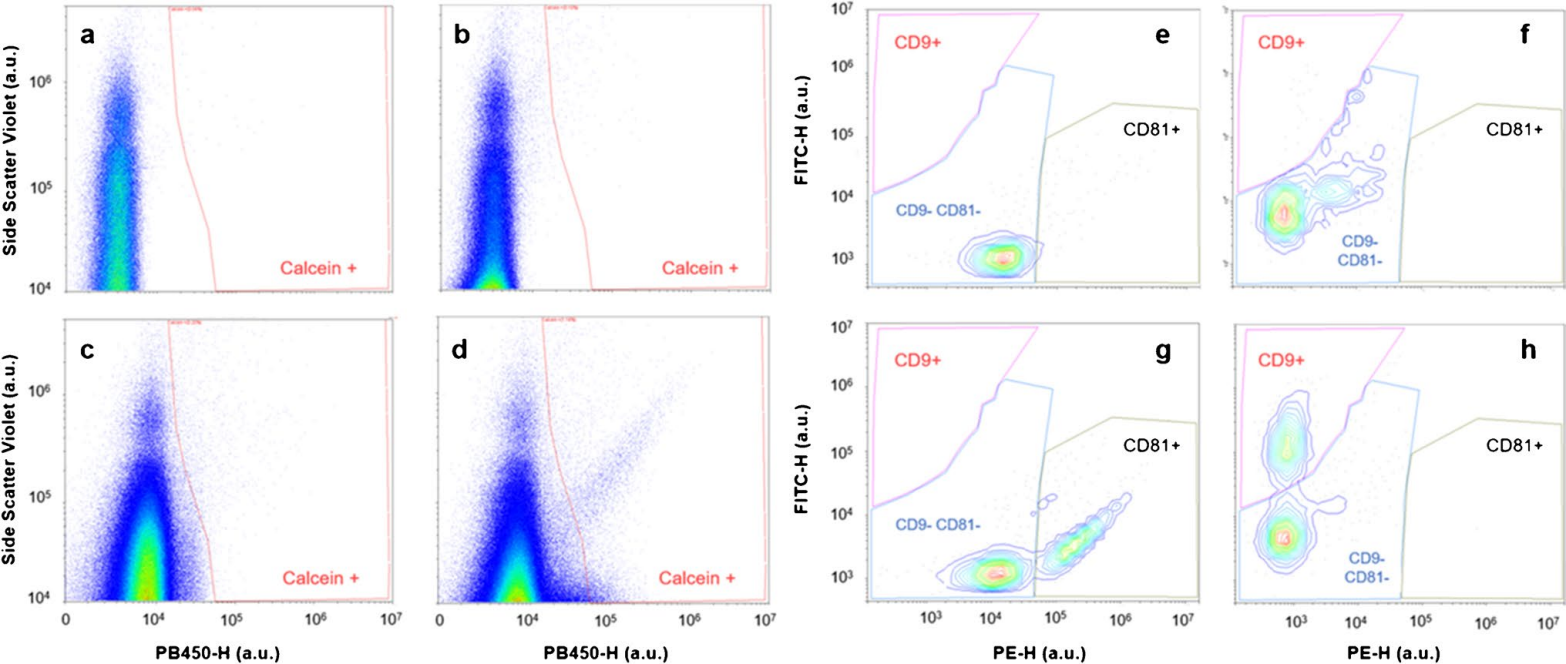
Cytogram representing side scatter violet vs fluorescence detected by PB450 detector. **a** Phosphate-buffered saline total events. **b** EV hUCESC sample 1-B total events. **c** Calcein-violet total events. **d** EV hUCESC sample 1-B incubated with calcein-violet total events. Cytogram represents the calcein-positive events in a FITC detector fluorescence vs PE detector fluorescence diagram. **e** Antibody anti-CD81. **f** Antibody anti-CD9. **g** EVs incubated with antibody anti-CD81. **h** EVs incubated with antibody anti-CD9

A multiplexed exosome assay was prepared using double tagging with calcein and each specific exosomes’ antibody (CD9 or CD81). Calcein identifies metabolically active intact vesicles, which can transform the non-fluorescent dye into the fluorescent form, whereas specific anti-CD9 (or CD81) detects exosomes. Figure 3 shows the results for calcein-violet dye (detected with PB450 channel). As can be seen, PBS and EVs have no fluorescence signal (Fig. 3 a and b); meanwhile, calcein produces a shift to higher fluorescence in channel PB450 (Fig. 3c). When the EVs were incubated with calcein, two populations were found in the calcein-positive window, one with a greater side scatter violet (SSCviolet) than the other. These results agree with Lucchetti’s study [26]. This calcein-positive window was selected for the analysis with the specific antibodies.

The results of the analysis of exosomal specific surface biomarkers were represented plotting FITC fluorescence (fluorophore for anti-CD9) vs PE fluorescence (fluorophore for anti-CD81). As can be seen in Fig. 3 e and f, blanks for both analyses were measured for setting the CD9 negative CD81-positive window. Then, the incubation with CD81 (Fig. 3g) and CD9 (Fig. 3h) was measured obtaining a CD81/CD9-positive population. Finally, an isotype control with an antibody anti-IgG with a double tag with both FITC and PE was assayed, and there were no unspecific unions of this antibody to the exosomes. This assay proves the presence of exosomes in our hUCESC-EV samples. More cytometry results are summarized in Fig. S5 and in a previous publication [27].

### New approximation to estimate EV concentration using a calibration curve

**Fig. 4:**
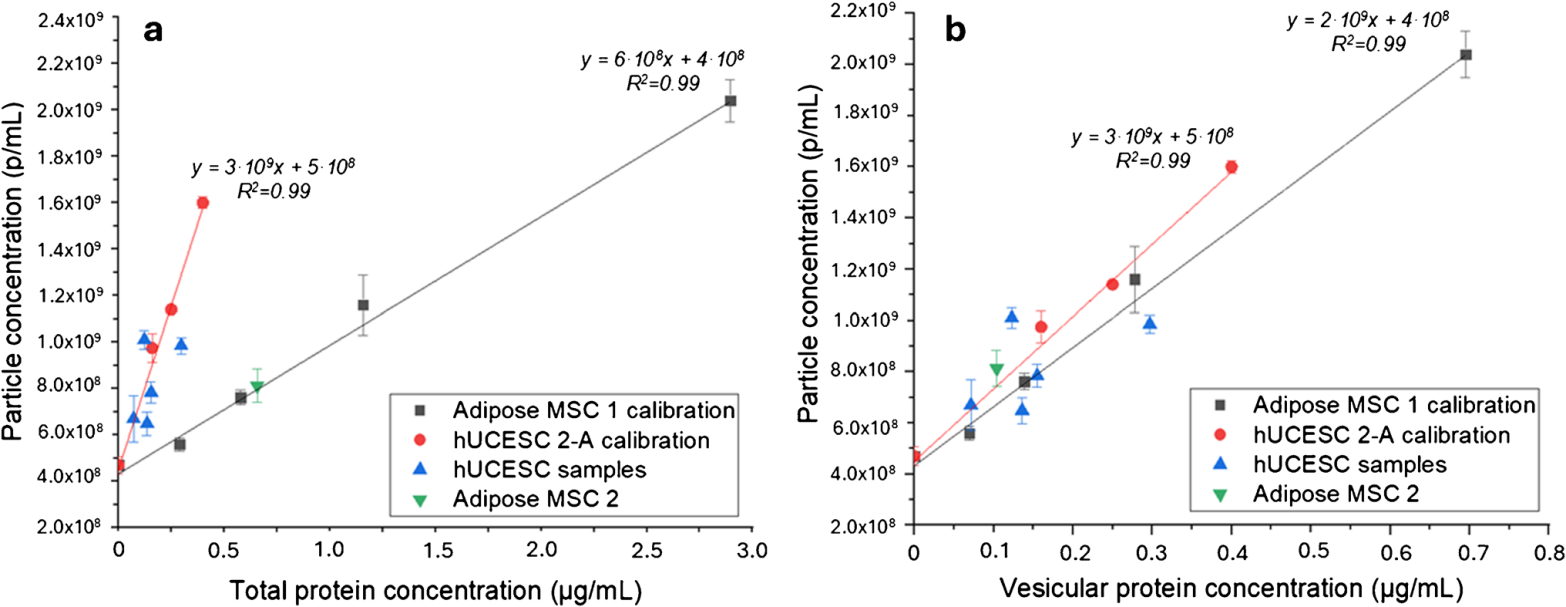
Calibration curves: **a** particle concentration (particles mL.^−1^) vs total protein concentration: hUCESC 2-A (red-circle) and adipose MSC 1 (black-square). **b** Particle concentration vs vesicular protein concentration: hUCESC 2-A (red-circle) and adipose MSC (black-square). hUCESC samples 1, 2-B, 3, 4, 5 (blue-triangle) and adipose MSC 2 (green-triangle)

As said before, NTA is the most widely used technique for determining the size and particle concentration of EVs, but it requires some operational skills for the adjustment of software settings and operation, a rigorous cleaning protocol for avoiding contamination and expensive equipment that may not be available to all laboratories. Here, a calibration curve based on particle concentration against EV protein concentration is proposed as an alternative method to estimate the particle concentration. Based on our purity analysis and previous studies [24], when the purity of the EV sample is high or its purity can be determined (i.e., by HPLC-SEC), vesicular protein can be used to calculate particle concentration. With this idea, two calibration curves were assayed by measuring serial dilutions of samples of different origins: hUCESC 2-A sample and adipose MSC 1. As can be seen in Fig. 4a, the slopes of the calibration curves are significantly different. Using the hUCESC 2-A calibration curve, the particle concentration for the hUCESC samples (1, 2-B, 3, 4,) were calculated, and the results are in good agreement with those obtained by NTA. However, the particle concentration obtained using the adipose MSC calibration curve differs greatly than the concentration obtained by NTA. This could be due to the presence of contaminants in the adipose MSC which has lower purity than the hUCESC samples as can be seen by the SEC results and the ratio of particles per µg of protein. As protein purity was impacting the quality of the adipose MSC calibration curve, a protein concentration correction was undergone.

Using the protein purity calculated by SEC, the vesicular protein content of the EVs from adipose MSC was recalculated, and a new calibration curve (particles mL^−1^ against vesicular protein in µg mL^−1^) *y* = 2·10^9^*x* + 4·10^8^ was obtained (Fig. 4b), which presents a similar slope to the calibration curve from hUCESC 2-A (*y* = 3·10^9^*x* + 5·10^8^). Concentration (particles mL^−1^) for four dilutions of the samples was calculated by both vesicular protein calibration curves, and the inter-assay variations were calculated. As can be observed in Table 4, inter-assay variations for hUCESC 1 and 2-A are similar (< 20%) to those of the same test made by NTA, while for adipose MSC, they are even inferior to the NTA results (< 35%). Additionally, an ANOVA test was performed to check if there are differences between the results found by NTA and the calibration curves, and no differences were found (*p* > 0.05).

**Table 4:** Particle concentration estimation using the developed calibration curves (particles mL^−1^ vs vesicular protein concentration (µg mL^−1^)) compared with NTA and their inter-assay variations (NTA commercial value = 4.4 × 10^11^ particles mL^−1^)

*EV sample*	*NTA concentration (p/mL* × *10*^*11*^*)*	*hUCESC 2 calibration*		*Adipose MSC calibration*	
		*Concentration (p/mL × 10*^*11*^)	*Inter-assay RSD (%)*	*Concentration (p/mL × 10*^*11*^)	*Inter-assay RSD (%)*
*Adipose MSC 1*	4.08	4.78	20	4.13	35
	11.2	13.6*		12.1*	
	7.62	4.29		3.68	
	5.80	6.24		5.45	
*hUCESC 1*	1.51	1.82	20	1.61	20
	1.52	1.34		1.16	
	1.43	1.34		1.16	
	1.94	1.18		1.02	
*hUCESC 2-A*	0.98	0.93	18	0.82	18
	0.75	0.93		0.82	
	0.64	0.63		0.55	
	0.73	0.75		0.66	

*Outlier rejected by Dixon’s *Q* test

Another advantage about this calibration is that BCA measurements have lower inter-assay variations than NTA (see Table S6). These results showed that the calibration curve using commercial EVs can be used to estimate the particle concentration of EVs with similar precision to NTA (see Table S7). It is important to highlight the necessity to evaluate the vesicle purity for obtaining an accurate measurement of particle concentration and this information must be included in the commercial standards or reference materials.

## Discussion

Cell-free therapy using EVs, a promising substitute for stem cell therapy, has applications in immune disorders, regenerative medicine, and drug delivery. The application of new EV treatments requires highly effective subpopulation isolation and characterization procedures as well as a complete understanding of their composition. However, the research on EVs is currently limited due to the lack of standardized and validated methods for isolation and characterization to have comparable laboratory results. Moreover, both the heterogeneity of EVs and the choice of instrument settings may cause an appreciable analytical variation. Additionally, well-characterized EV reference standards are necessary to validate the isolation and characterization methodologies, but nowadays, the so-called commercial “standards” lack proper characterization as it has been proved in this manuscript.

When analyzing the vesicles size distribution, NTA reproducibility could be hindered due to the limitations of the technique. First, the intrinsic polydisperse nature of the samples hampers the detection of light scattering techniques like NTA. This technique does not detect particles below 50 nm [17, 18] which is in accordance with our results (minimum size around 50 nm). However, EVs below 50 nm, called exomeres, require a bigger centrifugal force like 167,000 g to sediment by UC [28, 29]. This means that only vesicles above 50 nm are expected in our samples, and this is also supported by our TEM results (Figs. 1 and S2).

Inter-variations of 15–18% measured by NTA for particle concentration analysis of hUCESC samples were similar to those reported before [18, 30]. However, inter-variations were increased for adipose MSC exosomes (43%) which could be due to impurities like proteins, lipoproteins, or aggregates present in the commercial EVs from MSC origin. It has been proved that proteins can scatter light and be erroneously identified by NTA as a particle [19]. Another explanation of this phenomenon is that variations could be due to EV stability, because changes on EV size due to aggregation over time were observed with the NTA camera. Moreover, NTA measurement is heavily affected by the presence of microvesicles, and their detection could severely impact the particle concentration and, especially, the size distribution. NTA cannot differentiate between vesicles and similar-sized lipoproteins or protein aggregates, which could easily lead to an inaccurate enumeration of EVs. This fact is more critical when EVs are isolated from samples with high protein content like human serum or milk rather than FBS-depleted secretome which could explain the irreproducibility found in the adipose MSC samples. In addition, the preparation of the samples must be done just before the measurement to minimize EV aggregation with time. These results show that although NTA is considered a powerful characterization technique for EV particle concentration, it has limitations due to the sample nature, size, dilution, equip accessibility, and price. The significant size dependence of the technique makes the dispersed light intensity of very small particles disappear below the background noise. Also, large particles tend to be overestimated because these particles scatter more light than the smaller ones. Thus, NTA can clearly cause overestimation of large particles and underestimation of the particle number which could affect results in Table 2.

Protein assays are subject to interference by certain molecules that alters the correct value of the result. Regarding the quantification of vesicular protein, measurements of protein impurities co-isolated with EVs also can affect the accuracy of EV-protein quantification, as can be seen by the total protein discrepancies shown in Table 3. This especially might be problematic with low purity samples as interferences by lipids, free proteins, and lipoproteins produce erroneously high values for total protein assays. In this work, protein content estimated for hUCESC 4 sample by BCA and Bradford assays agreed closely which means that hUCESC 4 sample presents no BCA lipid interference; meanwhile, the Bradford protein concentration for serum 2 sample was lower than the value obtained by BCA assay. These results suggest the presence of lipids in serum 2 which interfere with the BCA protein assay. This is in accordance with HPLC-SEC results because serum 1 has peaks at low retention times which could be from lipoproteins co-isolated from the serum. In fact, when calculating protein purity from the HPLC-SEC areas, both commercial adipose MSC and serum EVs are between 20 and 25%. These results show that total protein assays are not a reliable way of vesicle determination or normalization. Because of this, complementary protein assays and techniques like HPLC or asymmetrical field flow fractionation (AF4) to separate contaminations are required to accurately determine EV concentration based in vesicular protein concentration. This work shows the necessity to certificate the protein and lipid purity of the commercial “standards” and to develop methods to check purity and determinate vesicle concentration. This work probed that BCA produce an overestimation of total protein in serum EVs due to lipid/lipoprotein contamination. Because of this, the total protein in standards and samples should be also determined by another assay not interfered by lipids, like Bradford. This study is important because lipids/lipoproteins could interfere in the protein purity evaluation by HPLC-SEC.

To study the free-protein contamination, the developed HPLC-SEC can clearly discriminate pure vesicle preparations from those with contaminating proteins. The EV purity can be estimated from the corresponding peak areas, and only vesicular protein should be considered to calculate particle concentration. However, it should be highlighted that protein aggregates or other non-vesicular entities with similar size to EVs are not easily separated by this chromatography. However, NTA measurements possess these same limitations, and it is a problem that scientists should work to solve.

Finally, the methodology developed in this work based on a calibration curve to estimate the vesicle concentration is a fast, precise, low-cost, and easy to perform technique. The method is a good alternative to conventional NTA and FC methodologies which are more time-consuming and need high-cost equipment and qualified people to perform the measurement.

## Conclusions

Although interest in EVs is growing fast, implementation of EVs in therapeutic applications requires their validated isolation and characterization. Their application is hampered by limitations in isolation and purification technologies and nomenclature, as well as in the capacity to determine size, concentration, and molecular content. This limits the interpretation and comparison of the research conducted on exosomes.

The first limit is the lack of an appropriate nomenclature for reporting EV research. Even if “Minimal Information for the Studies of Extracellular Vesicles” (MISEV) statements are the recommended nomenclature between experts, there is a clear misuse of the term exosomes in the literature. This hinders a proper bibliographic comparison of results between authors because the term “exosomes” is often used even when there is a lack of proper sample characterization in the work. Moreover, the lack of a standardized isolation method limits the interpretation and comparison of the research conducted on exosomes. Even using the golden standard for EV isolation, differential ultracentrifugation, differences in protocols may lead to different results. In addition, strong ultracentrifugation force may lead to partial EV aggregation and lysis.

In addition to the heterogeneity of the EV population, the presence of contaminants (protein complexes and lipoproteins) in biological and clinical EV samples should never be overlooked. The development of efficient and reliable exosome isolation and purification methodologies is needed in order to further advance in EV’s field.

Another limitation is the lack of good, standardized protocols for EV characterization. The use of protein content to estimate the amount of EV requires the determination of EV purity. This information must be included in commercial EVs. Moreover, the development of EV reference materials is required for the standardization and validation of these techniques.

This study shows that a limitation in NTA is that differences in dilution and measurement time affect the reproducibility and accuracy of the technique. In this work, an alternative new methodology to determine the particle concentration using a calibration curve which was constructed from EVs with calculated purity is proposed. The results were similar to these obtained by NTA and even better reproducibility than NTA. This methodology is a low cost and fast tool to estimate the particle concentration.

In conclusion, the promising therapeutic applications of these vesicles are a new hope in Biomedicine, but a previous optimal and complete analytical characterization should simplify the subsequent biomedical studies. Commercial EVs should display the protein and/or lipid purity and also the type of protein assay used to check the possible matrix interferences.

## Supplementary Information

Below is the link to the electronic supplementary material.

ESM 1 (DOCX 1.22 MB)

## Data Availability

No datasets were generated or analysed during the current study.

## Abbreviations

BCA: Bicinchoninic acid assay
BSA: Bovine serum albumin
CD: Cluster of differentiation
CM: Conditioned media
DLS: Dynamic light scattering
DMEM: Dulbecco’s modified eagle’s medium
EVs: Extracellular vesicles
FITC: Fluorescein isothiocyanate
hUCESC: Human uterine cervix stem cells
MISEV: Minimal information for the studies of extracellular vesicles
MSC: Mesenchymal stem cells
NTA: Nanoparticle tracking analysis
PBS: Phosphate- buffered saline
PE: Phycoerythrin
PVDF: Polyvinylidene fluoride
SSC: Side scatter
TEM: Transmission electron microscopy
TFF: Tangential flow filtration
TSG101: Tumor susceptibility gene 101
