# Supplementary material for: Limitations and challenges in the characterization of extracellular vesicles from stem cells and serum
Source: Mikrochim Acta. 2025 Apr 21;192(5):311. doi: 10.1007/s00604-025-07147-4 (PMC12011935; doi:10.1007/s00604-025-07147-4)
Supplement: Supplementary file 1 — (DOCX 1.22 MB) [file 604_2025_7147_MOESM1_ESM.docx]

**Limitations and challenges in the characterization of Extracellular Vesicles from stem cells and serum**

**Authors:** Sara Escudero-Cernuda^1^, Noemi Eiro^2^, María Fraile^2^, Francisco J. Vizoso^2*^, Belén Fernández-Colomer^3^ and María Luisa Fernández-Sánchez^1*^

**Address:**

1. Department of Physical and Analytical Chemistry, University of Oviedo, Oviedo, Spain.

2. Research Unit, Jove Hospital Foundation, Gijón, Spain.

3. Service of Neonatology, Department of Pediatrics, Hospital Universitario Central de Asturias, Oviedo, Spain.

***Corresponding authors:**

María Luisa Fernández-Sánchez

Department of Physical and Analytical Chemistry, University of Oviedo.

Avda. Julián Clavería, 8, 33006 Oviedo, Asturias, Spain.

Phone: +34 985103071

e-mail: marisafs@uniovi.es

Francisco J. Vizoso

Research Unit, Jove Hospital Foundation.

Avda. Eduardo Castro, 161, 33920 Gijón, Asturias. Spain.

Phone: +34 985320050 Ext. 84216

e-mail: investigacion@hospitaldejove.com


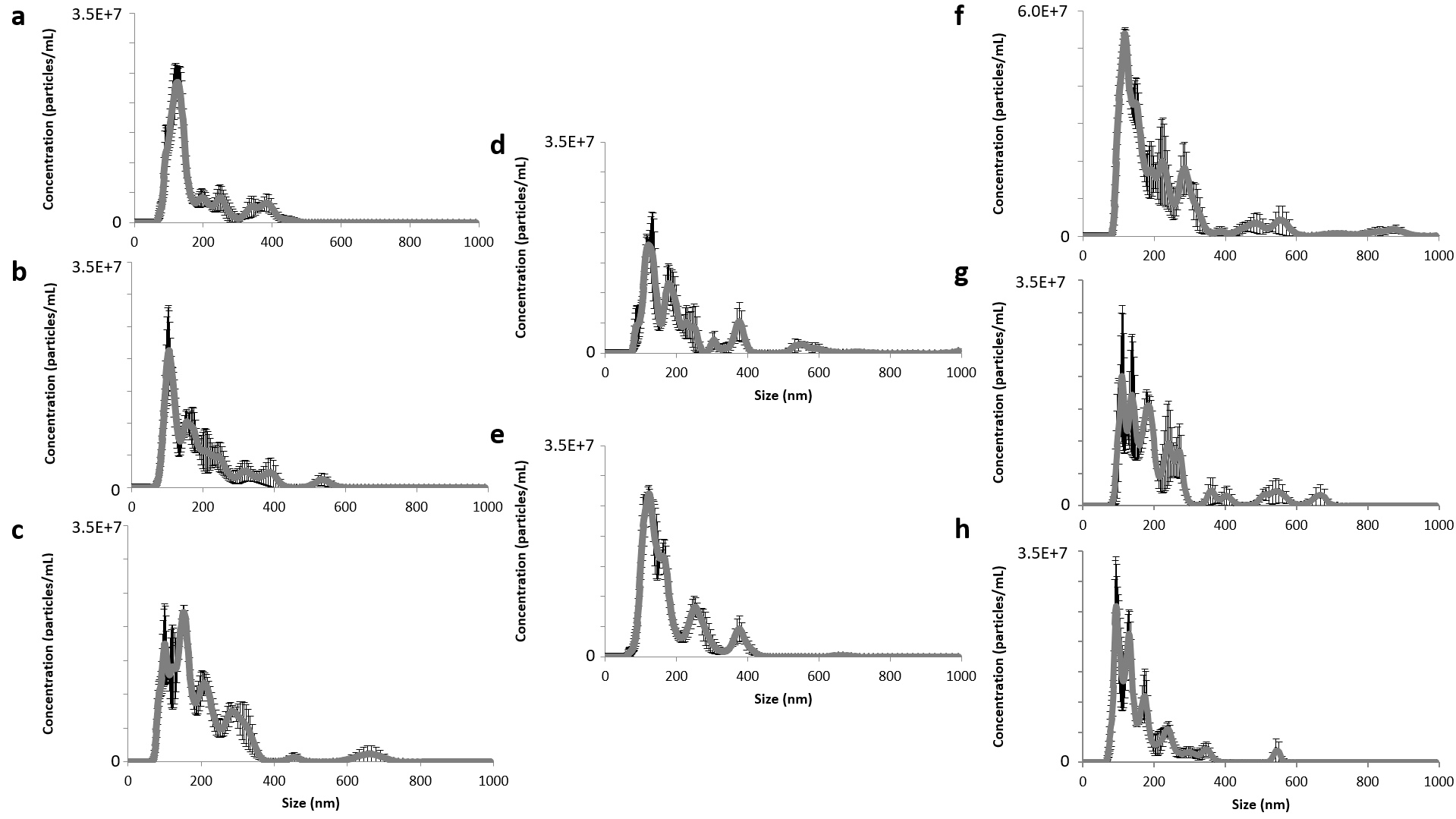


**Fig S1** Size dispersion graphs calculated by NTA. **(a)** hUCESCs 1, **(b)** hUCESCs 2-A, **(c)** hUCESCs 2-B, **(d)** hUCESCs 3, **(e)** hUCESCs 4, **(f)** Adipose MSC 1, **(g)** Adipose MSC 2, **(h)** Serum


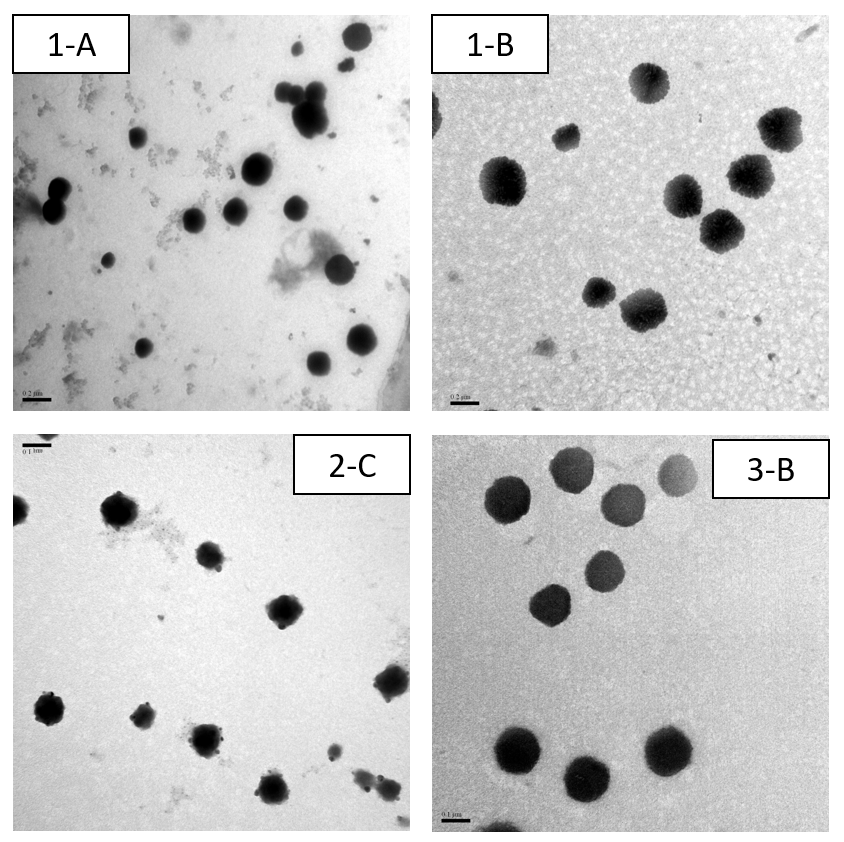


**Fig S2** TEM representative images of samples hUCESC 1-A and hUCESC 1-B with scale of 200 nm and hUCESC 2-C and 3-B with scale of 100 nm


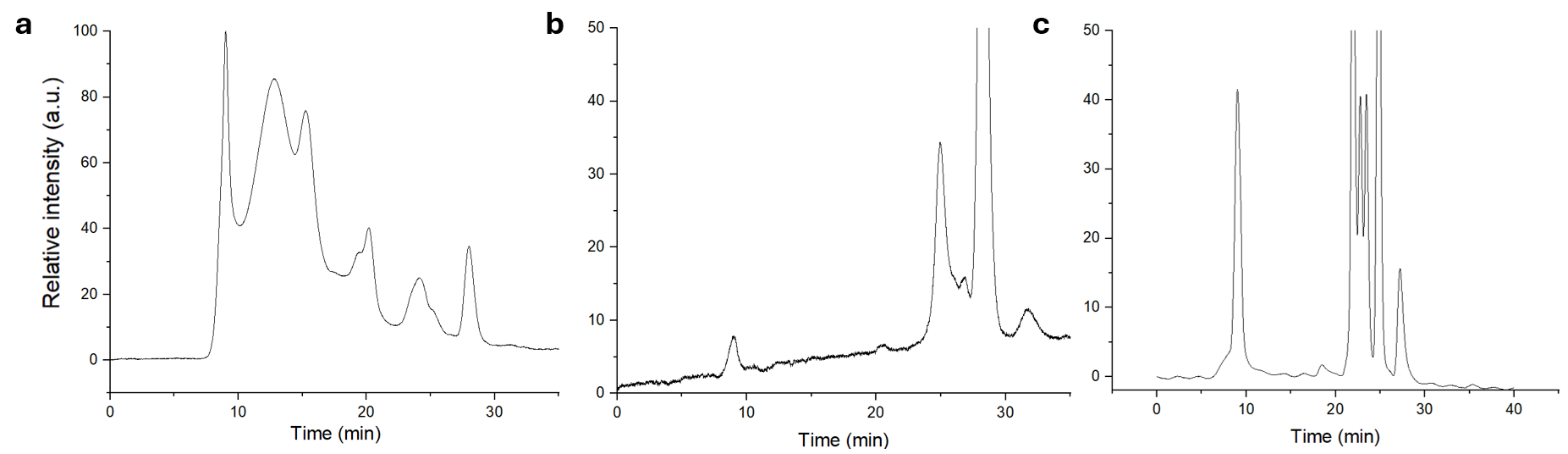


**Fig S3** EVs protein purity study by SEC-HPLC-UV/VIS. **(a)** Serum 2, **(b)** hUCESC 4, **(c)** hUCESC 5-B


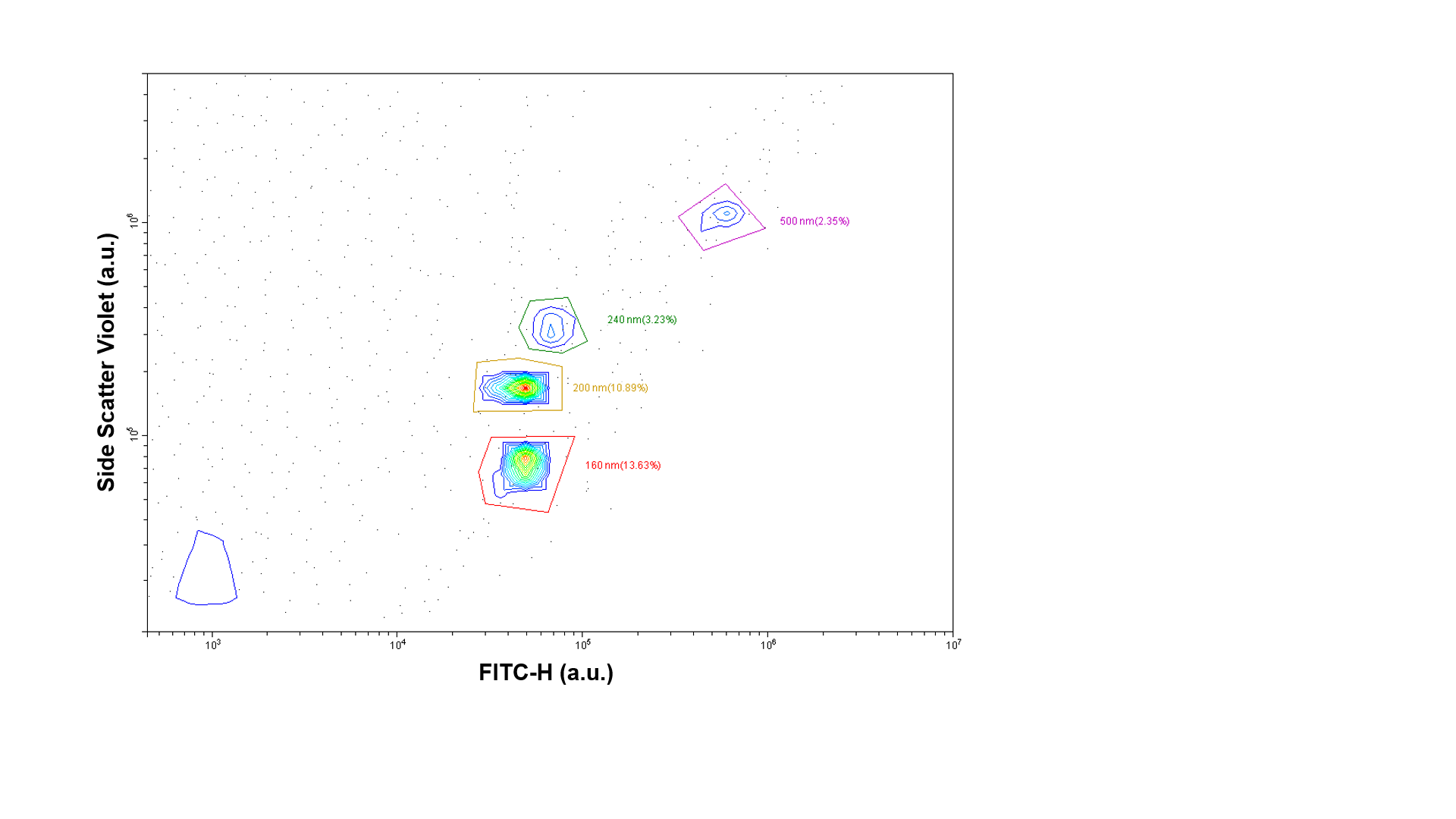


**Fig S4** Cytogram of the optimized separation of Megamix-Plus SSC fluorescent Beads


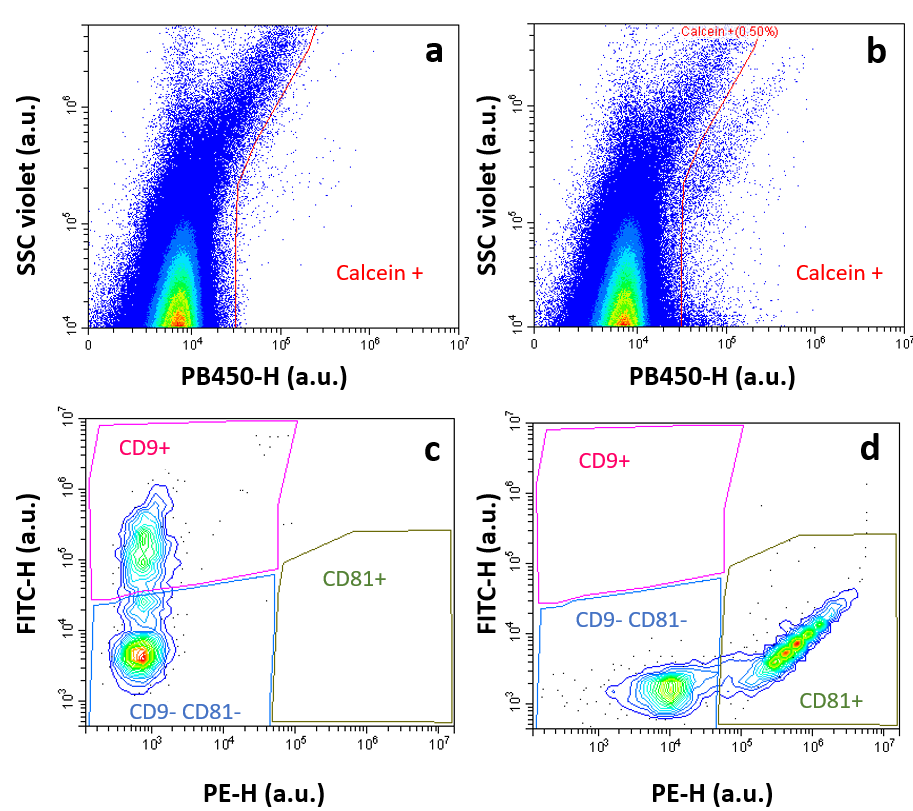


**Fig. S5** Flow Cytometry analysis of hUCESC 2-C. **(a)** Calcein control, **(b)** EVs incubated with calcein, **(c)** EVs incubated with antibody anti-CD9, **(d)** EVs incubated with antibody anti-CD81

**Table S6** Dilution in chronological order, size distribution, median intra- and inter-assay RSD and total protein intra-assay RSD for EVs Adipose MSC 1 commercial standard (NTA commercial value= 4.4x10^11^ p mL^-1^) and hUCESCs EVs Samples by NTA

| **EVs Sample** | **Dilution** | **Median size (nm)** | **Median Intra-assay RSD (%)** | **Median Inter-assay RSD (%)** | **Total protein Inter-assay RSD (%)** |
| --- | --- | --- | --- | --- | --- |
| Adipose MSC 1 | 1:200 | 137 ± 3 | 2 | 12 | 7 |
|  | 1:2000 | 125 ± 3 | 2 |  |  |
|  | 1:100 | 142 ± 19 | 13 |  |  |
|  | 1:500 | 116 ± 3 | 3 |  |  |
| hUCESCs 1 | 1:200 | 122 ± 3 | 2 | 13 | 2 |
|  | 1:100 | 129 ± 6 | 5 |  |  |
|  | 1:100 | 117 ± 4 | 3 |  |  |
|  | 1:68 | 147 ± 4 | 3 |  |  |
| hUCESCs 2-A | 1:100 | 135 ± 3 | 2 | 7 | 3 |
|  | 1:100 | 121 ± 7 | 6 |  |  |
|  | 1:40 | 122 ± 2 | 2 |  |  |
|  | 1:64 | 130 ± 2 | 2 |  |  |

**Table S7.** Comparison of NTA concentration determination (p/mL) with the developed calibrations of hUCESC 2-A and Adipose MSC-1 for particle concentration determination.

| ***EVs Sample*** | ***hUCESC 2-A calibration (p/mL · 10^11^)*** | ***Adipose MSC-1 calibration (p/mL ·10^11^)*** | ***NTA (p/mL·10^11^)*** |
| --- | --- | --- | --- |
| *hUCESC 1* | 1.82 | 1.61 | 1.51 |
| *hUCESC 2-B* | 0.93 | 0.82 | 1.01 |
| *hUCESC 3* | 0.82 | 0.73 | 0.65 |
| *hUCESC 4* | 0.86 | 0.76 | 0.99 |
| *hUCESC 5* | 1.79 | 1.58 | 1.34 |
| *Adipose MSC 2* | 7.72 | 6.84 | 8.13 |
